# SPR and SPR Imaging: Recent Trends in Developing Nanodevices for Detection and Real-Time Monitoring of Biomolecular Events

**DOI:** 10.3390/s16060870

**Published:** 2016-06-14

**Authors:** Mihaela Puiu, Camelia Bala

**Affiliations:** 1 R&D Center LaborQ, University of Bucharest, 4-12 Regina Elisabeta Blvd., Bucharest 030018, Romania; elenamihaela.puiu@g.unibuc.ro; 2 Department of Analytical Chemistry, University of Bucharest, 4-12 Regina Elisabeta Blvd., Bucharest 030018, Romania

**Keywords:** surface plasmon resonance imaging, microfluidics, lab-on-a-chip, point-of-care-testing

## Abstract

In this paper we review the underlying principles of the surface plasmon resonance (SPR) technique, particularly emphasizing its advantages along with its limitations regarding the ability to discriminate between the specific binding response and the interfering effects from biological samples. While SPR sensors were developed almost three decades, SPR detection is not yet able to reduce the time-consuming steps of the analysis, and is hardly amenable for miniaturized, portable platforms required in point-of-care (POC) testing. Recent advances in near-field optics have emerged, resulting in the development of SPR imaging (SPRi) as a powerful optical, label-free monitoring tool for multiplexed detection and monitoring of biomolecular events. The microarrays design of the SPRi chips incorporating various metallic nanostructures make these optofluidic devices more suitable for diagnosis and near-patient testing than the traditional SPR sensors. The latest developments indicate SPRi detection as being the most promising surface plasmon-based technique fulfilling the demands for implementation in lab-on-a-chip (LOC) technologies.

## 1. Introduction

The last two decades have witnessed the outstanding breakthrough of surface plasmon resonance (SPR) technology in clinical diagnosis, environmental monitoring, drug discovery, and polymer engineering, covering a broad area of health and biological sciences [1,2,3]. The main strong points of the SPR assays lay in their non-invasive detection and real-time monitoring of binding events, such as antibody–antigen, protein-protein, enzyme-substrate or inhibitor [4,5], protein-DNA, receptor-drug, protein-polysaccharide [5,6], protein-virus [7], and living cell-exogenous stimuli [8,9]. Additionally, SPR detection allows direct measurements of affinity and kinetic constants of biomolecular interactions, and does not require fluorescence or radioactive labelling of biomolecules (which is notable, as such forms of labelling may impair binding) [10,11]. Since the introduction of SPR technology by Biacore in Sweden in 1990, the applications in the area of biosensor, lab-on-a-chip (LOC), and point-of-care (POC) have experienced an accelerated growth, reflected by the published papers, reaching a peak within 2008–2010 [7,12]. The performances of the SPR sensors in terms of instrumentation, data processing, and analysis appear to be fully exploited and developed in the last six years, yet reaching a plateau, probably due to the rise of localized surface plasmon resonance (LSPR) [12] and SPR imaging (SPRi) assays [13]. These SP (surface plasma)-based optical techniques offer much higher sensitivity and facile extension to a highly multiplexed architecture than conventional SPR [4,14]. In this paper we will review the SPR techniques—conventional and SPR imaging—with emphasis on the innovative aspects of their *modus operandi* and design over the last decade (2006–2016). The ability of these techniques to address the challenges associated with the implementation in diagnosis and near-patient testing will be critically discussed. Additionally, the SPR sensor configuration from prism to optic fiber coupling with nanostructures for local field enhancement [11], the modulation type, and the possibility to exploit the surface acoustic waves (SAW) mixing in a microfluidic system to reduce the long lasting periods of the SPR bioassays, will be also underlined.

## 2. Basic Principles of SPR-Based Techniques

Most SPR sensing devices are developed following a common pattern: optical sensor, microfluidic chips, sampling system, data acquisition/analysis software [2,15]. The sample delivery at the sensor surface is ensured usually through continuous flow for kinetic measurements in order to prevent mass transport limitations and stopped flow for affinity assays [15,16]. The SPR sensor is sensitive only to media near the chip surface (up to 300 nm) [4,17,18], therefore the design of the microflow cell plays a crucial role in achieving well-controlled, reproducible sample delivery and significantly reduced sample volume [12]. A rigorously controlled isothermal regime should also be maintained since the temperature fluctuations greatly influence not only the SPR sensor response, but also the kinetics and the affinity of the biomolecular interactions [19].

The basic components of the SPR sensor are a light source and its optical system, optical coupling components (a prism, grating, waveguide, or optical fiber), an imaging optical system, and a photodetector [12,20]. Starting from the configuration of the coupling components and type of light wave modulation, various formats were recently developed for both SPR and SPRi approaches to improve the sensitivity and resolution, as will be shown further.

The terms “surface plasmons” and “polaritons” are assigned to some quasi-particles exhibiting wave-particle duality (as the photons and the phonons), which primarily exist on the surface of substances containing abundant free electrons, or metals [17]. Plasmon refers to the oscillation of free electrons density with respect to the fixed positive ions in a metal. The term “surface plasmon” designates the charge density wave propagating along the metal’s surface [21]. The interaction of photons hitting the metal surface with the surface plasmons yield the so-called surface plasmon polaritons (SPPs), which are entangled quasi-particles, composed of both surface plasmons and photons [10]. The wavelengths of photons vary with the medium refractive index n through dispersion, but their oscillation frequencies remain unchanged. For a quasi-particle, the dependence of the propagation wavelength λ on its oscillation frequency ω is given by:

(1) $$\lambda =\frac{2\pi c}{\omega }$$

where *c* is the speed of light in a vacuum. Consequently, the wave propagation number β becomes:

(2) $$\beta =\frac{2\pi }{\lambda }$$

The dispersion of SPPs relates to the propagation number β of SPPs along the interface, with their oscillation frequency [22,23]. The simplest geometry sustaining SPPs is that of a flat interface between a metal (1) and a dielectric medium (2) with the dielectric constants ε_1_ (complex) and ε_2_ (real), respectively [10,13,24]. Only a p-polarized electromagnetic (EM) or transverse magnetic(TM) wave is able to sustain SPPs. The electromagnetic field of a SPP at a metal-dielectric surface interface is obtained by solving Maxwell’s wave equations in each medium, incorporating the suitable boundary conditions at the metal–dielectric medium interface. The latter refers to the continuity of the tangential components of the electric and magnetic fields across the interface and to the disappearance of these fields infinitely far from the interface [2,23,25]. Therefore, the propagation number of the SPPs along the interface $\beta =2\pi /\lambda_{x}$ is related to their oscillation frequency via the two dielectric constants of the interfacing metal–dielectric medium ε_1_ and ε_2_, (for a given oscillation frequency of incident light ω) [17,19].

(3) $$\beta =\frac{\omega }{c}\;\sqrt{\frac{\varepsilon_{1}\varepsilon_{2}}{\varepsilon_{1}+\varepsilon_{2}}}$$

The intensity of the SPPs’ EM field undergoes a rapid decrease as the distance from the interface increases, and the SPP excitation itself is confined in the near-field. Following the interaction with the incident light, the SPPs absorb the energy from the incident photons and propagate along the surface with the wave propagation number β. Thus, the intensity of the reflected beam is less than the intensity of the incident and, consequently, the resulting reflectance (R) is less than unity. The amount of the absorbed energy dissipated inside the metal by the damping of electrons is strongly dependent on the optical and material properties in the near-field. The SPP waves display maximum intensities that decrease exponentially with the distance in both media, with a variable penetration from 100 nm to 600 nm (for visible VIS and near infrared NIR wavelengths) [25].

There is a specific angle at which SPPs can be excited and resonated (the SPR angle) for a given monochromatic light source. At settled optical and material conditions, the SPR angle relies only on the refractive index of the medium (or dielectric constant) [21,22]. The refractive index (RI) of the contacting dielectric medium is the one of the most sensitive variables correlated with near-field phenomena, such as transport, temperature variation, evaporation, chemical reactions, or ligand-receptor binding [4,5,10]. For a SPR sensor, the sensitivity is defined by the ratio of the change in sensor output to the change in the quantity to be measured (the refractive index), while the resolution defines the smallest change in the refractive index that produces a detectable change in the sensor output. The magnitude of sensor output change that can be detected depends on the level of uncertainty of the sensor output—the output noise [26]. The limit of detection (LOD) achieved with the SPR technology is estimated as 1 pg·mm^−2^ of bound biomaterial at the sensor surface [15]. This sensitivity is sufficient for bioassays involving high molecular weight compounds, such as antibodies, proteins, or DNA [3,7,27]. Still, the sensitivity needs to be improved for low molecular weight targets (typically less than 500 Da), such as mycotoxins, drugs, vitamins, *etc.*, as well as for larger low copy number targets, such as, for example, bacteria and viruses, which are pathogenic even in ultra-low quantities [9]. The sensitivity and LOD of conventional SPR sensors can be increased by using nanostructured substrates and gold nanoparticles (GNPs) modified tags [11].

## 3. Signal Modulation for SPR and SPRi Sensors

**Figure 1:**
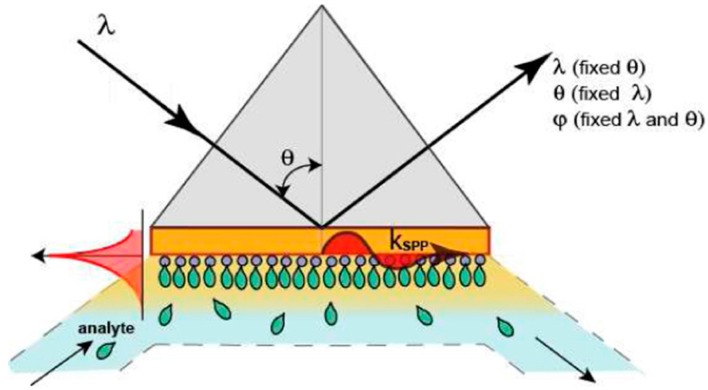
Interrogation modes for commercial surface plasmon resonance (SPR) instruments (reproduced from [30] with permission of OSA Publishing).

According to their modulation approach, SPR devices can be classified into four categories: angle modulation, wavelength modulation, amplitude modulation, and phase modulation [28,29], as depicted in Figure 1.

The main problem of current commercially available SPR instruments arises from the fact that LOD is conditioned by the level of noises in measurements and is usually estimated as 10^−6^–10^−5^ Refractive Index Units (RIU), for devices based on angular, wavelength, and amplitude interrogations [15,30,31,32].

### 3.1. Amplitude Modulation

This type of modulation is performed at a fixed incidence angle and wavelength, with the RI variation being detected on the basis of the change in the resonance intensity. The drawback of this approach is given by the low sensitivity and resolution, because the output noise and resolution increase with the noise generated by the light source [31]. This is generally the case of the SPRi systems based on amplitude modulation, measuring the reflectivity of monochromatic incident p-polarized light at a fixed angle, unlike the scanning angle SPR or scanning wavelength SPR (traditionally termed “SPR spectroscopy”) [19]. In this respect, the spectroscopic SPR sensors outperform the SPRi sensors. The contribution of the light source noise in SPR imaging can be substantially reduced by referencing [26,31]. The SPRi sensors use 2D detectors to measure the variations of the intensity of the reflected light (expressed as the percent reflectivity, % R) [13].

The changes of the chemical composition or of the layer thickness near to the metallic surface, inducing variations in the local dielectric constants, are recorded as image contrast. The biomolecular events are detected by collecting difference images, obtained by subtracting a reference image from a post-binding image [19]. Because they are based on intensity interrogation, the SPRi sensors suffer from one order of magnitude worse resolution than the conventional SPR sensors (10^−6^ compared to 10^−7^ RIU, respectively) [13,26].

### 3.2. Angular Modulation

This is the most used type of modulation, based on the identification of the angle at which the SPR occurs and is characteristic for the prism configuration. The metallic film surface is irradiated with monochromatic light and scanned for a certain range angle. There the angular scanning is achieved (a) using a scanning source and (b) using a rotating light source or prism with a light at a specific angle. For a fixed source, the beam of light has a divergent angle [12,31].

### 3.3. Wavelength Modulation

Here, the sensing principle is based on fixing the angle of the incident light at a certain value and modulating the wavelength of the reflected light. The resonant condition is achieved in a prism configuration through attenuated total reflection (ATR). The reflected intensity dip is measured *versus* the change in the refractive index over a range of incident wavelengths [33].

### 3.4. Phase Modulation

Under SPR, the phase of light can cause a sharp dip in the angular dependence of the phase on the p-polarized light. This method introduces the “probe” beam and the “reference” beam, the latter which is used for comparison with the s-polarized portion of the main beam [30]. The phase shifts Δφ due to interference are observed through spatial displacement of the light beam. The phase shift in SPR conditions ∆φ_max_ produces a change in the refractive index **n** of the medium, so that the phase derivative ∆φ/∆n can be measured. [34]. Maximal phase variations occur in the very dip of the SPR curve, where the vector of the “probe” electric field is maximal, whereas maximal amplitude changes are observed on the resonance slopes; thus, the sensitivity of the phase to RI variations is at least 10 times larger than the sensitivity of amplitude to RI changes [34,35]. Phase noises are orders of magnitude lower compared to amplitude ones, providing a better signal-to-noise ratio [30]. The phase modulation is better fitted for SPRi and multiplex analysis with parallel detection of thousands of channels [36]. On the other hand, recently developed phase-modulation systems can achieve a LOD of 4 × 10^−8^ RIU [34,36] and are suitable for incorporation into SPRi devices, but due to their complexity, they are not amenable for point-of-care testing (POCT) platforms [26,31].

## 4. Configuration of SPR and SPRi Sensors

### 4.1. Prism Coupling

**Figure 2:**
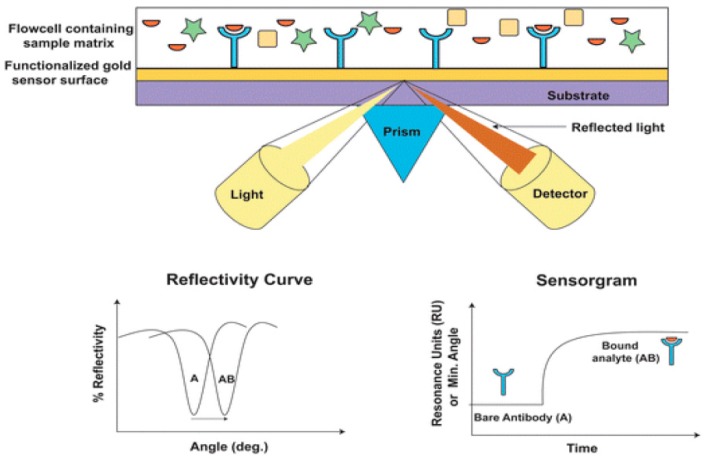
Operating principle of conventional SPR sensor based on Kretschman configuration: the target binding to the immobilized receptor (**up, center**) causes a time-variation of the refractive index of the medium near the surface which is monitored by the shift of the resonance angle (**below, left**) or resonance wavelength (**below, right**) (reproduced from [24] with permission of RSC Publishing).

The most common approach to produce excitation of surface plasmons in sensors is prism coupling in attenuated total reflection (ATR) conditions, using Kretschmann geometry. According to this configuration, a thin metal film (typically silver or gold with 50 nm of thickness) is deposited directly on top of the glass prism surface [5,12,20]. The incident light, after total internal reflection (TIR), generates an evanescent wave (EW) that penetrates the metal film and excites the surface plasmons at the interface between the sample and the metal film. The principle of the spectroscopic SPR bioassays is based on time-monitoring of the changes in the refractive index of the medium near the metal surface, caused by the target binding to the surface immobilized receptor. These changes entail time-variation of the resonance wavelength of the incident light (at fixed resonant angle) and time-shifting of the resonance angle at a fixed wavelength (Figure 2).

The main advantage of this type of modulation remains the fact that, since the angle of incidence is continuously monitored, the kinetics of binding events can be recorded in real-time (although this information is not essential for a POC testing). Still, it was recently reported that there was an angular interrogation type sensor used for a 10-min detection of the anti-dengue virus in human serum samples [37]. Here, four dengue virus serotypes were used as receptors on a biochip using only 1 μL serum sample. Following the angle modulation, the ratio of each dengue serotype in samples was determined with 83%–93% sensitivity and 100% specificity. This result may envision angular SPR sensors as amenable for POCT, even if portable/hand-held instruments are still not available [38].

**Figure 3:**
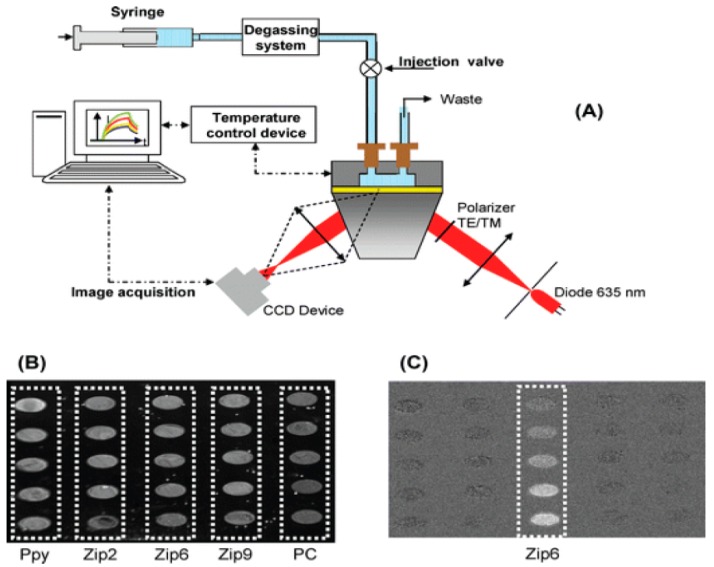
(**A**) Experimental set-up of a SPR imaging (SPRi) sensor. The recorded data are represented as intensity variation of the reflected light at a fixed angle for each region of interest (ROI) selected; (**B**) Image of the DNA chip as seen from the charge-coupled device (CCD); (**C**) Differential image registered during the injection of a single-strand ssDNA target. The image was obtained by subtracting the image registered before injecting the target from image of the chip during hybridization (reproduced from [39] with permission of RSC Publishing).

Most SPRi devices adopt the Kretschman configuration, using a plane-polarized light with fixed angle as incident light, and a charge-coupled device (CCD) camera for the detection of the reflected light [28]. This allows the visualization of the whole biochip in real time. If the sensor surface is split into multiple sensing spots, then this multi-array format is able to simultaneously monitor hundreds of bioreceptor/target bindings with the parallel support of a digital image, representing the intensity of binding in a color scale (Figure 3) [19,20].

The measurement areas (the regions of interest, ROI) can be accurately selected through direct image control in order to identify and reduce the non-specific binding. Thus, spots without receptors or spots on gold can be used as negative control surfaces. Currently, the spot surfaces lie within 50 μm^2^ to 1 cm^2^, and they can be created either manually or by automatic spotters [19]. One key feature of the SPRi sensors is the versatility in designing various DNA-immobilization formats on gold surface for both detection and genetic analysis [40,41]. Moreover, the real-time monitoring of DNA hybridization allows the estimation of the kinetic and equilibrium constants of DNA duplex formation using a simple DNA microarray chip [42,43]. While most SPRi assays are carried out in isotherm conditions, temperature scans from room temperature to 70 °C were first introduced to SPRi by Fiche [40] to monitor DNA melting in real time, thus estimating the enthalpy and the entropy of the hybridization. The analysis of the thermal stability of DNA duplexes on biochips and the detection of single-point mutations were performed recently from the DNA melting curves obtained on a DNA grafted gold SPRi chip [43,44].

### 4.2. Grating Coupling

**Figure 4:**
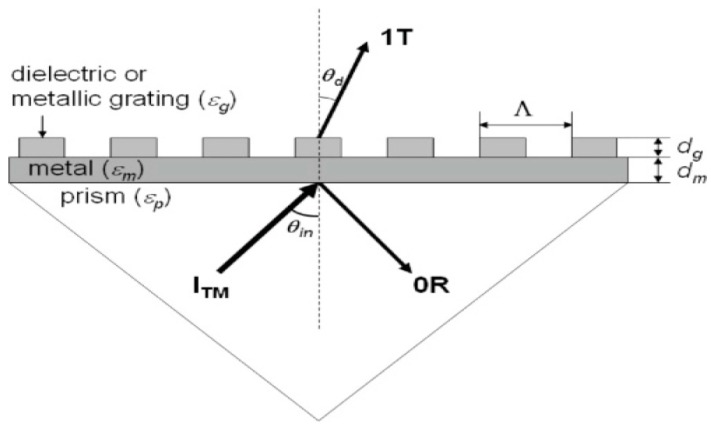
Grating coupling configuration of a SPR sensor with angular modulation. The thin metal layer deposited onto prism has a thickness d_m_. The metallic or dielectric grating has a period Λ and a fill factor f. The diffracted wave is transmitted in a substrate environment (water or air) (reproduced from [45] with permission of OSA Publishing).

According to this configuration, the incident light beam propagates on a metallic diffraction grating with periodic corrugation; consequently, the light is multi-staged diffracted. The diffraction waves at different levels correspond to different propagation angles and modes. The surface plasmon excitation occurs when the wave vector of a diffraction wave at one level is coupled with the surface plasmon waves SPWs [12]. A grating coupling configured SPR sensor with angular interrogation is presented in Figure 4.

The metallic grating that converts the surface plasmons into radiation modes induces an increase of the interaction area, which supports the excitation of SPs and mediates the interaction between excited plasmons and local events on a perturbed metal surface. For practical sensing applications, the real-time detection of gaseous or aqueous variations can be accomplished by measuring the diffraction characteristics in an enhanced transmission mode [45]. The disadvantage is that the colored samples are prone to interferences since some colored solutions can absorb radiation from the visible spectrum. Grating coupling structures able to excite the SP and simultaneously to disperse the diffracted light to the detector array were used to detect short nucleotides with the lowest resolvable concentration of 200 pM and a resolution of 3 × 10^−7^ RIU [26,46]. Another compact multichannel SPR sensor based on angular spectroscopy of SP diffraction grating was reported to detect short nucleotides [47]. The sensors displayed chip-to-chip reproducibility of the assay, with a resolution of 6 × 10^−7^ RIU and the lowest detectable concentration of 1 nM. It appears that grating coupling structures incorporated in compact frames without the use of a spectrometer are the key for implementation of SPR or SPRi sensors in lab-on-a-chip technologies. Recently, a surface acoustic wave (SAW)-enhanced SPR microfluidic biosensor in which SAW-induced mixing and phase-interrogation grating-coupling SPR are combined in a single lithium niobate lab-on-a-chip has been reported [48,49]. In this assay the adsorption of thiol-polyethylene glycol and the binding kinetics of avidin/biotin were monitored. A considerable shortening of the analysis time was achieved, owing to the fluid mixing enhancement by means of the SAW-generated chaotic advection [48]. Thus, a reduction of the time saturation binding kinetics of 82% and 24% for polyethylene and avidin adsorption, respectively, were obtained.

### 4.3. Optic Fiber Coupling

**Figure 5:**
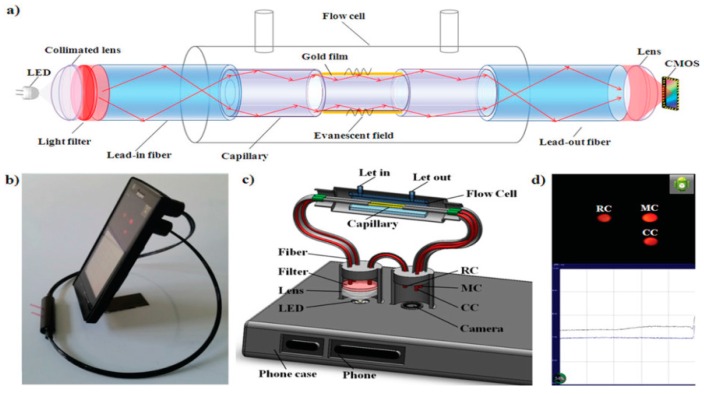
Schematic representation of a SPRi device coupled with a smart phone (**a**) SPRi cell with complementary metal-oxide-semiconductor (CMOS) camera and light emitting diode (LED) as a light source (**b**) photograph of the SPRi sensor installed on a smart phone (**c**) illustration of the opto-mechanical instrumentation (**d**) camera of the smart phone capturing images from the measurement, control, and reference channels (MC, CC and RC). This work is licensed under a Creative Commons Attribution 4.0 International License. The images or other third party material in this article are included in the article’s Creative Commons license, unless indicated otherwise in the credit line; if the material is not included under the Creative Commons license, users will need to obtain permission from the license holder in order to reproduce the material. Reproduced with permission from [54].

The guidance of incident light in optical fibers based sensors is also based on total internal reflection, but the prism is replaced by the core of an optical fiber. Generally, the silicon cladding from a small region of the fiber (at the end or in the middle) is removed and is coated with a metal layer, which is further surrounded by a dielectric sensing layer. When the wavelength modulation is used, a polychromatic light beam is launched into one of the ends of the optical fiber. Under TIR conditions, the generated evanescent field excites the SPs at the fiber core-metal layer interface [50]. These optic-fiber based SPR sensors operate using either wavelength or intensity interrogation of the sensing areas. The reported sensor resolutions are comparable for the two types of operations: 8 × 10^−5^ RIU and 5 × 10^−5^ RIU, respectively [12]. The fiber optic SPR (FO-SPR) sensor has advantages such as smart size, high resolution, flexibility, and miniaturization [28] which can allow sensing in harsh conditions. Moreover, the couplers lose less light and transmit signals over a long distance [12]. Beside detection, these features allow FO-SPR to operate at different temperatures in order to obtain the thermodynamic parameters of DNA hybridization at solid surface from the analysis of the Langmuir binding isotherms [51]. Recently, a FO-SPR platform was used for the detection of DNA mutations by real-time monitoring of DNA duplex melting during high resolution temperature cycling [52]. Here, polymerase chain reaction PCR amplified DNA from nine different serogroups of the bacterium *Legionella pneumophila* (a common human pathogen responsible for atypical pneumonia) was directly screened for single-point mutations exploiting the signal of the DNA melting enhanced by means of GNP labels. It is also worth mentioning the SPR sensor developed by Jang (detection of prostate-specific antigen in a sandwich immunoassay) which displayed a resolution of 2.5 × 10^−6^ RIU [53], and the SPRi sensor based on optic fiber coupling developed by Yanase (monitoring the reaction of living cells attached to the fiber tip in real time, with a LOD of 1.65 × 10^−3^ RIU) [8]. Interestingly, optic fiber SPRi sensors seem better fitted for assays supporting clinical diagnosis, not only for their ability to be incorporated in compact and multiplexed platforms, but also because conventional SPR sensors detect only an average of RI changes in the presence of thousands of cells and provide only a small number of sensing channels (<10). Moreover, conventional SPRs are not able to reveal the intracellular distribution of RI [9]. Another miniaturized compact SPRi sensor based on a smart phone platform was recently reported, where the light-weight optical components and sensing element are connected by optical fibers to a phone case (Figure 5) [54]. A smart application was used to extract the light intensity information from the camera images; the light intensities of each channel were recorded every 0.5 s with refractive index changes.

The performance of the smart phone-based SPRi platform was evaluated by monitoring the binding of bovine immunoglobulin G (IgG) to the surface immobilized Staphylococcal Protein A (SPA). The reported resolution was 7.4 × 10^−5^ RIU and the LOD for IgG was 47.4 nM.

## 5. Localized Surface Plasma Resonance (LSPR)

**Figure 6:**
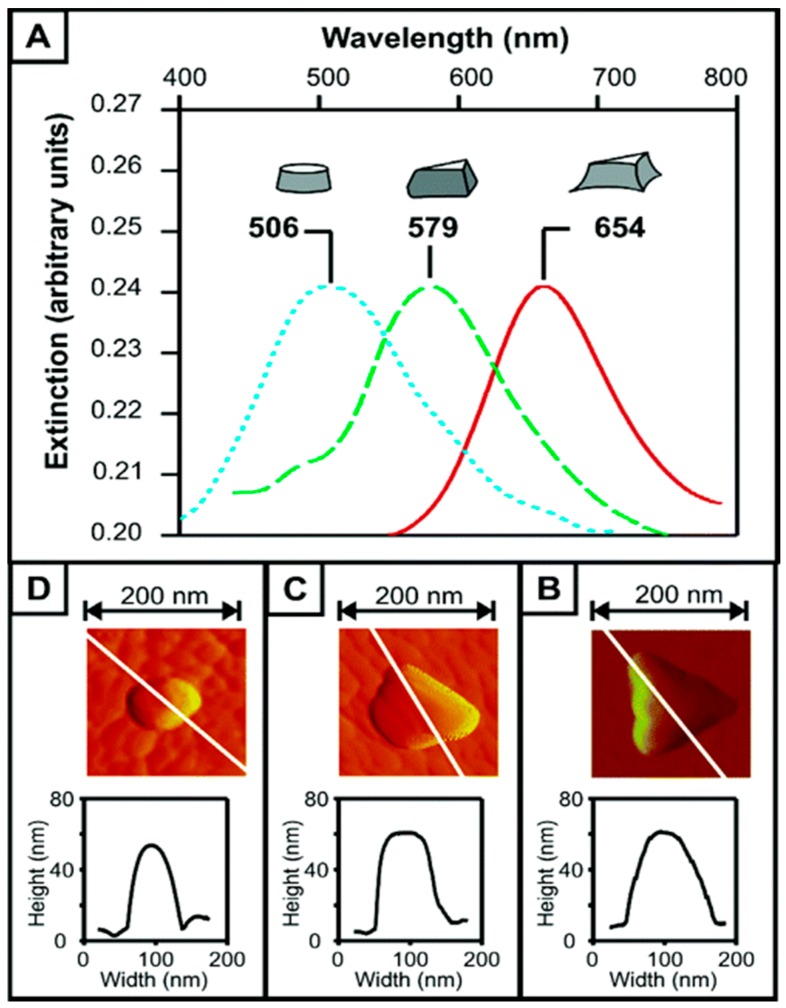
Shifting of the localized surface plasma resonance (LSPR) absorption bands following the shape modification of silver nanoparticles (NPs) (reproduced from [57] Copyright (2005) American Chemical Society). (**a**) Towards shorter wavelengths after subsequent chronocoulometry measurements; (**b**) Atomic-force-microscope image following two chronocoulometry runs, (**c**) following one chronocoulometry run (**d**) before any electrochemical oxidation.

Surface plasmon-based transducers are generally divided into two categories: SPR, referring to the charge density oscillations propagating along a metal/dielectric interface, and localized SPRs (LSPRs) occurring on nanoscopic metallic structures (spheres, disks, holes, rods, shells, *etc.*). LSPR is the coupling of light into the resonance oscillation of charge density on the nano-structured surface. It appears as an intense absorption band over the spectral range [55]. Gold and silver nanoparticles (NPs) exhibit LSPR at visible as well as near-infrared frequencies, with sharp peaks in their spectral absorbance. The new generation of SPR devices incorporating these metallic nanostructures has the benefits of small foot-print for POC detection, ease of being integrated into an array format, and low cost for one-time disposal [56]. They also overcome two major drawbacks of the SPR assays: first, they are not temperature sensitive, because LSPR sensing is based on a simple absorbance measurement and can be performed using the common laboratory equipment; secondly, LSPR can significantly decrease the mixing time, since the sample spreads faster to the surfaces of the nanoparticles than to the metallic film [29,35]. The absorption wavelength of the LSP is characteristic of the type of material and is strongly dependent on the dielectric environment, but particularly to the size and shape of the NPs [12]. An example is offered in Figure 6 where the peak shift of the absorbance *versus* the shape modification of silver NPs can be observed. Different shapes can also be achieved with the nanoparticles of silver, aluminium, and other metals. Most methods to produce nanoparticles are based on lithography, including nanoimprint lithography, nanosphere lithography [57], electron beam lithography, focused ion beam lithography [11], and dip-pen lithography [12,14]. All of these methods have advantages and disadvantages and should be selected depending on the characteristics of the transducers and the assay formats.

One major disadvantage, however, is that LSPR sensors are prone to interference because they respond not only to refractive index variations but also to non-specific binding. These interactions can severely compromise the measurement of the target in complex matrices, and hence limit the applicability and impact of the sensor [58].

## 6. Nanostructured Transducers Amenable for Miniaturized SPR Devices

Since SPR occurs following the interaction of light with a metal, the performance of SPR-based sensors in terms of sensitivity and resolution are mainly correlated with the characteristics of the metallic surface (thickness, structure, *etc.*). Especially for SPRi sensors, when high quality and high contrast in the image are required, attention was paid to reduce the background resonance by patterning the metallic thin film with regularly repeating micro- and nanostructures. Micro- and nano patterned arrays are functioning as sources of SP, confining the SPR inside the micro- and nanowells [59]; they also enclose supported lipid bilayer membranes [60], lipid vesicles [59], scaffold for pore-spanning lipid membranes [61], and antibodies for cancer biomarkers [62]. A SPR sensor based on microhole arrays with the diameter equal to half the periodicity displayed enhanced sensitivity to RI (>3000 nm/RIU), with a 10^−6^ RIU resolution, and proved a LOD of 10 nM for IgG detection. The advantages of these microplasmonic materials were the ease of fabrication and the use of conventional SPR instrumentation without modifications [63]. Another promising approach using a nanostructured transducer reports an affordable low-noise surface plasmon resonance (SPR) device based on extraordinary optical transmission (EOT) in metallic nanohole arrays; the sensor quantified antigen/25-kDa single-chain antibodies binding kinetics at concentrations below 1 nM, and provided dissociation constants ranging from 200 pM to 40 nM. This nanohole-based SPR instrument was built around a standard microscope and a portable fiber-optic spectrometer. The measured resolution of this platform was 3.1 × 10^−6^ RIU, without on-chip cooling, which is among the lowest reported for SPR sensors based on EOT [64]. Nanostructured SPRi sensors can also extract information on binding kinetics and affinity from several parallel microfluidic channels composed of nanohole arrays [11]. The target receptors are arrayed on gold through robotic spotting, microfluidics, or micro-contact printing. The recently developed techniques of SPR-phase imaging (PI) and nanoparticle-enhanced SPR-PI can perform multiplex affinity analysis of proteins and nucleic acids. SPR-PI utilizes a light emitting diode LED light source in near-IR region along with a wedge depolarizer to produce phase-dependent grating on single-strand ssDNA microarray [65]. The phase shift is measured in real time, allowing the detection of the bioaffinity adsorption. The target binding produces an increase of RI at the interface from the adsorbed nanoparticle causing a sharp phase shift. The efficiency of SPR-PI was demonstrated in two parallel experiments utilizing two different ssDNA aptamer microarray having different Langmuir adsorption coefficients to detect thrombin at variable concentrations [65]. Several recent SPR assays which are promising for implementation in POCT platforms are summarized in Table 1.

## 7. Conclusions

The overwhelming number of studies dedicated to SPR assays using metallic NPs, with various shapes and sizes, reveal the huge impact of these structures on the sensitivity and resolution of the SPR devices. The possibility of designing a multiplexed analysis format using nanostructured arrays, combined with different coupling and interrogation modes, and finally with non-invasive detection, has revived SPR and SPRi technologies. Probably, not very far in the future, we will see miniaturized SPR/LSPR/SPRi analyzers for near-to-patient applications arriving on the market. The major disadvantage of SPR sensing assays lies in the interference of non-specific bindings to the outcome signals. Furthermore, the kinetic and affinity parameters obtained with SPR sensors of the biomolecular events are not relevant for POCT. There are few works reporting SPRi detection on real samples, but in the future the combination of SPRi with mass spectrometry (MS) -structural identification of a molecule and the detection of various targets on a single chip may fulfil the tremendous demand for high-throughput analysis of the biomolecular events.

## Figures and Tables

**Table 1:** Analysis of biomolecular events with SPR for clinical diagnosis.

Platform	Key Features	Target	Ligand	Advantages	Matrix	Ref
Commercial SPR	Angular interrogation	Anti-dengue	Dengue virus	10 min detection	Serum sample	[37]
Biacore 3000		IgM	Serotypes	1 μL target solution required		
SPR	Angular interrogation	Anti-PA immunoglobulin G (IgG)	Protective antigen (PA) of anthrax toxin	Simple optical and mechanical design Low cost	Buffer	[64]
	extraordinary optical transmission (EOT) in metallic nanohole array					
SPRi	Angular interrogation	Anti-EGFR IgG	Membrane embedded epidermal growth factor receptor (EGFR)	Directly quantify the membrane embedded receptor expression level Ligand binding kinetics without the need of labelling	Buffer	[66]
SPRi	Angular interrogation	Human chorionic gonadotropin (hCG)	Anti-hCG IgG	Limit of detection (LOD) 45 ng/mL	Blood plasma	[67]
				LOD 100 ng/mL		
	Protein array	Activated leukocyte cell adhesion molecule (ALCAM)	Anti-ALCAM IgG	Simple optical and mechanical		
				design		
SPR-PI	LED light source in near-IR region along with a wedge depolarizer to produce phase dependent grating on single stranded DNA microarray	Thrombin	ssDNA aptamer	Simultaneous SPR biosensing and imaging	Buffer	[65]
				LOD 25 fM		
LSPR	Wavelength interrogation	Amyloid-beta-derived diffusible ligand (ADDL)	Anti-ADDL IgG	Highly selective, Uniform sensitivity Customized optical properties	Cerebro-spinal fluid	[68]
				High throughput label-free kinetic analysis		
				LOD 20 pM		

## Abbreviations

The following abbreviations are used in this manuscript:

SPR	surface plasmon resonance
LSPR	localized surface plasmon resonance
TIR	total internal reflection
CCD	charge coupling device
POCT	point-of-care testing
ATR	attenuated total reflection
LOD	limit of detection
EOT	enhanced optical transmission
SAW	surface acoustic wave
RIU	refractive index unit
PI	phase image
LED	light emitting diode
ROI	region of interest
TM	transverse magnetic
GNP	gold nanoparticle
IgG	immunoglobulin G
EW	evanescent wave
CMOS	complementary metal-oxide-semiconductor
PCR	polymerase chain reaction
MDPI	Multidisciplinary Digital Publishing Institute
DOAJ	Directory of open access journals
TLA	Three letter acronym
